# Identification and characterization of Dicer1e, a Dicer1 protein variant, in oral cancer cells

**DOI:** 10.1186/1476-4598-13-190

**Published:** 2014-08-13

**Authors:** Liliana P Cantini, Lourdes M Andino, Christopher C Attaway, Betsy Butler, Anca Dumitriu, Aaron Blackshaw, Andrew Jakymiw

**Affiliations:** Department of Oral Health Sciences and Center for Oral Health Research, Hollings Cancer Center, Medical University of South Carolina, 173 Ashley Avenue, Charleston, SC 29425 USA; Department of Pediatrics, Division of Hematology/Oncology, Medical University of South Carolina, 135 Rutledge Avenue, Charleston, SC 29425 USA

**Keywords:** Dicer1, Dicer1e, RNAi, EMT, miRNA, Protein variant, Oral cancer

## Abstract

**Background:**

The human *dicer1* gene has been predicted to produce several mRNA variants that encode truncated Dicer1 proteins of varying lengths. One of these Dicer1 variants, Dicer1e, was recently found to be differentially expressed in breast cancer cells. Because the expression and function of the Dicer1e protein variant has not been well characterized and the underlying molecular mechanisms for the development of oral squamous cell carcinomas (OSCCs) are poorly understood, the present study sought to characterize the biological role of Dicer1e and determine its relationship, if any, to OSCC pathogenesis.

**Methods:**

Western blot analyses were used to examine Dicer1e expression levels in a panel of oral cancer cells/tissues and during epithelial-mesenchymal transition (EMT), followed by 5′/3′-RACE analyses to obtain the full-length Dicer1e transcript. Biochemical fractionation and indirect immunofluorescent studies were performed to determine the cellular localization of Dicer1e and the effects of Dicer1e silencing on cancer cell proliferation, clonogenicity, and drug sensitivity were also assessed.

**Results:**

Dicer1e protein levels were found to be overexpressed in OSCC cell lines of epithelial phenotype and in OSCC tissues with its levels downregulated during EMT. Moreover, the Dicer1e protein was observed to predominantly localize in the nucleus. 5′/3′-RACE analyses confirmed the presence of the Dicer1e transcript and silencing of Dicer1e impaired both cancer cell proliferation and clonogenicity by inducing either apoptosis and/or G2/M cell cycle arrest. Lastly, Dicer1e knockdown enhanced the chemosensitivity of oral cancer cells to cisplatin.

**Conclusion:**

The expression levels of Dicer1e influence the pathogenesis of oral cancer cells and alter their response to chemosensitivity, thus supporting the importance of Dicer1e as a therapeutic target for OSCCs.

**Electronic supplementary material:**

The online version of this article (doi:10.1186/1476-4598-13-190) contains supplementary material, which is available to authorized users.

## Background

OSCC is a malignant neoplasm of the head and neck region accounting for over 90% of all subtypes of head and neck cancers [1]. Cancer of the oral cavity and pharynx are a significant global burden with an incidence of 400,000 new cases and more than 200,000 deaths worldwide [2, 3]. In the USA they represent 2.5% of the annually diagnosed malignancies in men [4] and it is estimated that more than 40,000 Americans will be diagnosed and approximately 8,000 will die [5]. Despite advances in the fields of oncology and surgery, the 5-year survival rate for all stages is approximately 62% and it has only modestly improved in the last 30 years [1, 5–7]. In order to develop new therapies for treating oral cancer, new molecular insights into its pathobiology are required.

RNA interference (RNAi) is a post-transcriptional gene regulatory mechanism that can precisely silence gene expression [8]. It is activated by exogenous small non-coding double-stranded RNAs (dsRNAs) or by endogenous, small non-coding RNAs known as microRNAs (miRNAs) [8, 9]. Human miRNAs play a regulatory role in diverse cellular and molecular processes including protection against viruses, responses to environmental conditions, cellular proliferation, differentiation, and apoptosis [10–12]. Because of the modulatory functions that they perform, it is not unexpected that the dysregulation of miRNAs or any protein related with their biogenesis, are implicated in the pathogenesis of human diseases such as cancer, fibrosis and immunologic disorders [13–18]. In this regard, it has been well documented that one of the key enzymes of the RNAi pathway, Dicer1, has an abnormal expression in different types of cancer, including OSCCs [15, 19, 20].

Dicer1 is a highly conserved multidomain RNase type III enzyme that plays an essential role in the RNAi and miRNA pathways [21, 22]. The human *dicer1* gene, which is located on chromosome 14, spans a region of about 71 kbp and comprises 29 exons [23, 24]. The gene encodes a 218-kDa protein that is found in almost all eukaryotes [9, 12, 25, 26]. Dicer1 is responsible for processing dsRNAs into small interfering RNAs (siRNAs) and precursor miRNAs (pre-miRNAs) into mature miRNAs [21, 27, 28]. The small non-coding RNAs generated by Dicer1 are typically between 20-27 nucleotides long [29, 30] and they function as a guide for the RNA-induced silencing complex (RISC) that targets mRNA for silencing [29, 31]. The targeting of the mRNA occurs through a base-pairing-dependent mechanism that leads to translational repression or mRNA degradation [8, 32, 33].

To date, a number of Dicer1 mRNA variants have been described; however, all the reported transcripts have been found to encode the same full-length protein because the diversity was observed to affect only the length and composition of either their 3′ or 5′-untranslated regions [27, 34, 35]. Recently, the first mRNA splice variant of the human *dicer1* gene bearing a modified coding sequence was identified in neuroblastoma cells [24]. In fact, the *dicer1* gene has been predicted to produce several mRNA splice variants in addition to the one found in neuroblastoma cells that encode truncated Dicer1 proteins of varying lengths [23]. One of these Dicer1 mRNA splice variants termed, Dicer1e, was predicted to translate a 93-kDa protein which was found to be differentially expressed between epithelial and mesenchymal breast cancer cells [36]. Because the expression and function of the Dicer1e protein variant has not been well characterized and it currently remains unclear as to its biological and pathological significance, this study sought to examine the biological role of the Dicer1e protein variant and determine its relationship, if any, to oral cancer pathogenesis.

## Results

### Dicer 1e is overexpressed in OSCC cell lines of epithelial phenotype and in OSCC tissues

**Figure 1:**
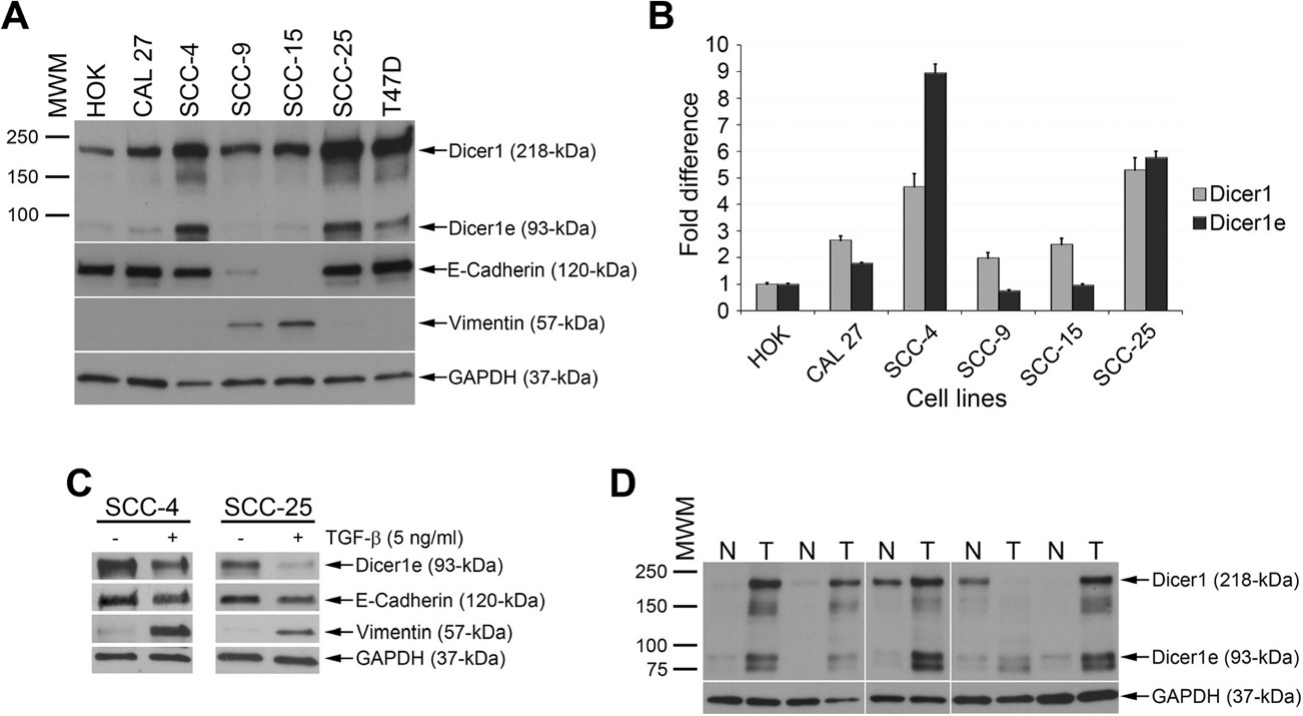
**Dicer1e is overexpressed in human OSCC cell lines of epithelial phenotype and in OSCC tissues. (A)** Western blot analysis of Dicer1 and Dicer1e expression in a panel of human OSCC cell lines (CAL 27, SCC-4, SCC-9, SCC-15 and SCC-25) compared to normal human oral keratinocytes (HOK). The breast cancer cell line T47D was used as a positive control for Dicer1e protein expression based on the findings from the Hinkal *et al*. study [36]. Epithelial and mesenchymal phenotypes were determined by examining the expression of E-cadherin and vimentin. GAPDH was used as a loading control. The data are representative of three independent experiments. MWM, molecular weight markers (kDa). **(B)** Quantitative measurement of the relative fold expression levels of Dicer1 and Dicer1e proteins in oral cancer cell lines compared to HOK. Dicer1 and Dicer1e protein levels were normalized to GAPDH. Values are expressed as mean ± SEM of three independent experiments. **(C)** Western blot showing Dicer1e expression in SCC-4 and SCC-25 cells stimulated for 8 days with TGF-β compared to unstimulated cells. Epithelial-mesenchymal transition was confirmed by examining the expression of E-cadherin and vimentin. GAPDH was used as a loading control. **(D)** Western blot analysis of Dicer1 and Dicer1e protein expression in human adjacent normal (N) and tumor (T) tissues. GAPDH was used as a loading control.

The human *dicer1* gene is predicted to produce several mRNA variants bearing modified coding sequences [23, 36], one of which, the 93-kDa Dicer1e protein variant, was reported to be differentially expressed in epithelial and mesenchymal breast cancer cells [36]. In order to determine the endogenous expression levels of Dicer1e in oral cancer cells, the expression of the ~93-kDa Dicer1e protein was examined in a panel of cell lines derived from tongue squamous cell carcinomas (SCCs) and compared to normal human oral keratinocytes (HOKs) by Western blot analysis (Figure 1A). Quantitation of the Dicer1e expression levels demonstrated that the OSCC cell lines (CAL 27, SCC-4, and SCC-25) of epithelial phenotype (high E-cadherin and low vimentin expression levels), exhibited approximately between 2 and 9-fold differences in Dicer1e protein levels compared to HOKs, whereas, OSCC cell lines of mesenchymal phenotype (high vimentin and low E-cadherin expression levels), exhibited either equivalent (SCC-15) or slightly reduced levels of Dicer1e expression (SCC-9, 0.8 fold) (Figure 1B). Together, these results corroborated the observed differential expression of Dicer1e in epithelial and mesenchymal breast cancer cell lines [36]. It is important to note that the Hinkal *et al.* study [36] also reported the differential expression of a 113-kDa Dicer1d protein variant in epithelial and mesenchymal breast cancer cells. However, in our analyses we did not detect a 113-kDa protein band in any of the oral cancer cell lysates. To verify that this was not due to differences between oral cancer cells and breast cancer cells, we also compared the Dicer1e migration patterns to that of the T47D breast cancer cell line that was similarly used in their study and found only evidence for the expression of the 93-kDa Dicer1e protein variant (Figure 1A). In addition to our analyses of Dicer1e protein levels, the expression levels of the 218-kDa Dicer1 protein were also analyzed/quantitated and observed to be upregulated in OSCC cell lines (Figure 1A and B), as previously reported [19]. Lower levels of Dicer1 were detected in mesenchymal cells compared with epithelial cells (Figure 1A and B), which was consistent with several other studies that have reported that the downregulation of Dicer1 expression levels also appear to be associated with epithelial-mesenchymal transition (EMT) [23, 36, 37].

Having observed a differential expression of Dicer1e in epithelial and mesenchymal oral cancer cell lines and knowing that Dicer1e protein levels have been reported to decrease during EMT using immortalized human epithelial mammary cells [36], we subsequently proceeded to analyze whether the induction of EMT in oral cancer cells would similarly affect Dicer1e protein levels. Stimulation of SCC-4 and SCC-25 cells with TGF-β, a known inducer of EMT in oral cancer cells [38, 39], was found to transition the cells from an epithelial to mesenchymal phenotype with a concurrent decrease in Dicer1e protein levels in comparison to unstimulated cells (Figure 1C). Thus, based on these results, it appeared that Dicer1e downregulation was associated with EMT.

The aberrant expression of Dicer1e protein observed in tongue SCC cell lines, prompted us to also examine the Dicer1e protein expression levels in five human tongue SCCs in comparison to their adjacent normal tissues. Consistent with our Western blot results for OSCC cell lines of epithelial phenotype, the ~93-kDa Dicer1e protein levels in five tissue sets were found to be overexpressed in all tongue SCC tissues compared with the adjacent normal tissues (Figure 1D). Additionally, the 218-kDa Dicer1 protein was also found to be upregulated in 4 of 5 OSCC tissue samples, thus, corroborating our previous findings that oral cancer tissues have increased levels of Dicer1 [19]. Of note, during our analysis of the human tissues, we also detected a ~75-kDa anti-Dicer1 antibody-reactive protein that was similarly upregulated in OSCC tissues. However, the identity of this protein was unclear and remains to be determined, as it did not correspond to any predicted Dicer1 variants previously reported by the Grelier *et al.* and Hinkal *et al.* studies [23, 36]. Nonetheless, these data suggested that in OSCCs the expression of Dicer1e protein was elevated compared to normal tissues.

### Oral cancer cells express the Dicer1e mRNA variant of the *dicer1*gene

**Figure 2:**
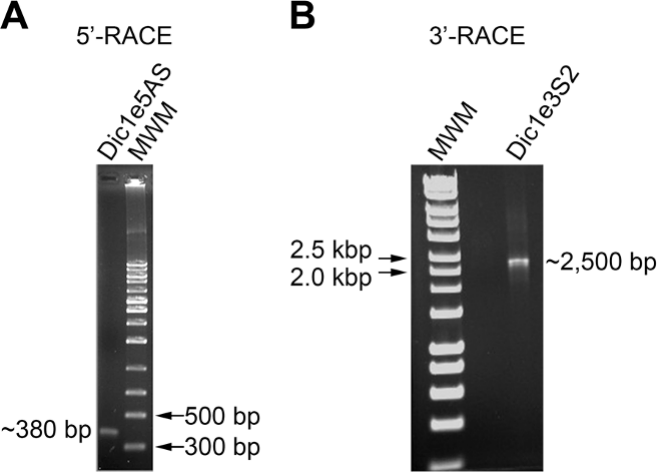
**5′ and 3′-RACE analyses confirm the presence of the Dicer1e transcript. (A)** 5′-RACE analysis using a reverse primer (Dic1e5AS) designed to target a unique sequence found only in Dicer1e resulted in the amplification of a ~380 bp product that closely corresponded to the expected 5′-RACE product size of 373 bp. **(B)** 3′-RACE analysis followed by nested PCR using a forward primer (Dic1e3S2) designed to target a unique sequence of Dicer1e resulted in the amplification of a ~2,500 bp product that closely corresponded to the expected 3′-RACE product size of 2,472 bp. MWM, molecular weight markers.

The human *dicer1* gene is located on chromosome 14 and spans a region of about 71 kbp and comprises 29 exons [24]. To date, a number of Dicer1 mRNA variants have been described; however, all the reported transcripts have been found to encode the same full-length protein [34, 35, 40]. More recently, however, the first mRNA splice variant of the human *dicer1* gene bearing a modified coding sequence was identified in neuroblastoma cells [24]. In fact, the *dicer1* gene has been predicted to produce 14 mRNA variants in addition to the one found in neuroblastoma cells, including 3 full-length forms and 11 mRNA variants that encode truncated Dicer1 proteins of varying lengths, one being Dicer1e [23, 36]. Although our data, plus the Hinkal *et al.* study [36], had confirmed that a ~93-kDa Dicer1 protein variant was present in cells, no biochemical evidence existed that this protein variant was the product of a predicted Dicer1 mRNA variant. As a result, to confirm that cells expressed the Dicer1e transcript, we performed 5′ and 3′-RACE analyses using mRNA harvested from SCC-25 cells, a cell line exhibiting one of the highest levels of Dicer1e protein expression (Figure 2). 5′-RACE analysis using a reverse primer (Dic1e5AS) designed to target a unique sequence found only in Dicer1e resulted in the amplification of a ~380 bp product that closely corresponded to the expected 5′-RACE product size of 373 bp (Figure 2A). Subsequent 3′-RACE analysis using a forward primer (Dic1/1e3S1), followed by nested PCR using a second forward primer (Dic1e3S2) designed to target the unique sequence of Dicer1e resulted in the amplification of a ~2,500 bp product that also closely corresponded to the expected 3′-RACE product size of 2,472 bp (Figure 2B). Cloning and complete sequencing of both the 5′ and 3′-RACE products confirmed the existence of the Dicer1e transcript. Of note, RT-PCR analysis was also performed in an attempt to detect the Dicer1d mRNA variant; however, no predicted PCR products were observed in either oral cancer cells or the T47D breast cancer cell line (data not shown). The absence of this transcript in cells corroborated our Western blot results and most likely explained why the 113-kDa Dicer1d protein was not expressed in cells.

**Figure 3:**
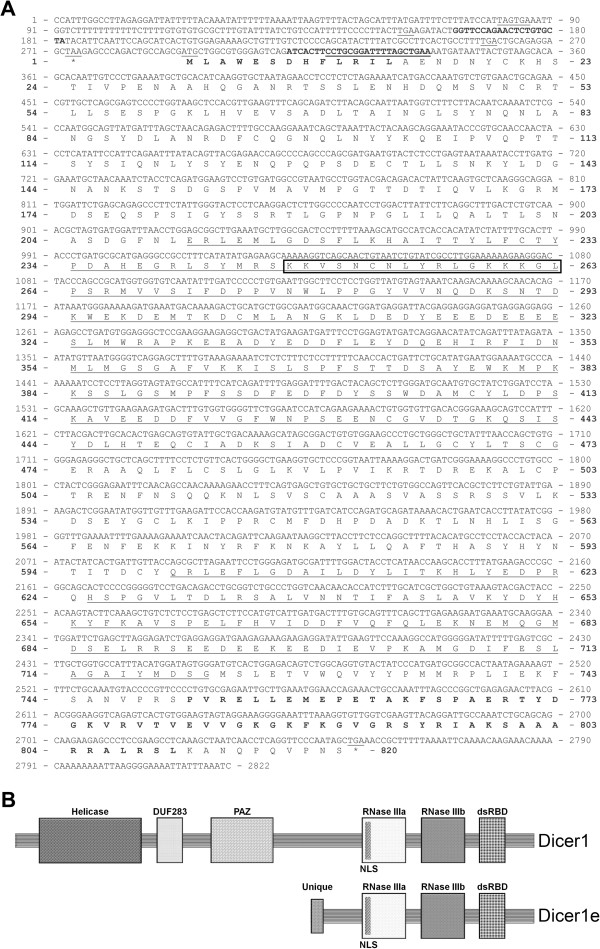
**Human Dicer1e. (A)** The Dicer1e cDNA sequence and its deduced amino acid sequence [GenBank: KJ175111]. The largest open reading frame encodes an 820 amino acid protein with a predicted molecular mass of ~93-kDa. The underlined nucleotides are the upstream in-frame stop codons, the translational methionine start site, the siDicer1e sense sequence, and the translational stop codon, respectively. The nucleotides in bold type represent the locations of the Dicer1/1e-specific sense primer (Dic1/1e3S1) and the Dicer1e-specific antisense/sense primers (Dic1e5AS and Dic1e3S2), respectively. The underlined amino acid residues are the two ribonuclease III (RNase III) domains. The amino acid residues highlighted by a box are the predicted nuclear localization signal (NLS). The amino acid residues in bold type represent a unique sequence of residues not present in the Dicer1 protein (Unique) and the double-stranded RNA binding domain (dsRBD), respectively. **(B)** Schematic representation of human Dicer1 protein in comparison to Dicer1e. Individual protein domain structures are indicated by different shadings. Helicase, ATPase/helicase; DUF283, domain of unknown function; PAZ, Piwi/Argonaute/Zwille.

The sequence of the complete Dicer1e cDNA and the predicted amino acid sequence [GenBank: KJ175111] are shown in Figure 3A. Comparison between the experimental and the NCBI AceView database (http://www.ncbi.nlm.nih.gov/IEB/Research/Acembly/) predicted cDNA sequences, demonstrated only minor differences in the 5′ and 3′-UTR regions with no alterations to the Dicer1e protein coding sequence. The Dicer1e transcript consisted of 2,822 nucleotides that were predicted to encode an 820 amino acid truncated protein form of Dicer1 (~93-kDa) comprising both RNase III domains, a nuclear localization signal (NLS) and the dsRNA binding domain (dsRBD) (Figure 3A and B).

### Dicer1e is predominantly localized in the nucleus

**Figure 4:**
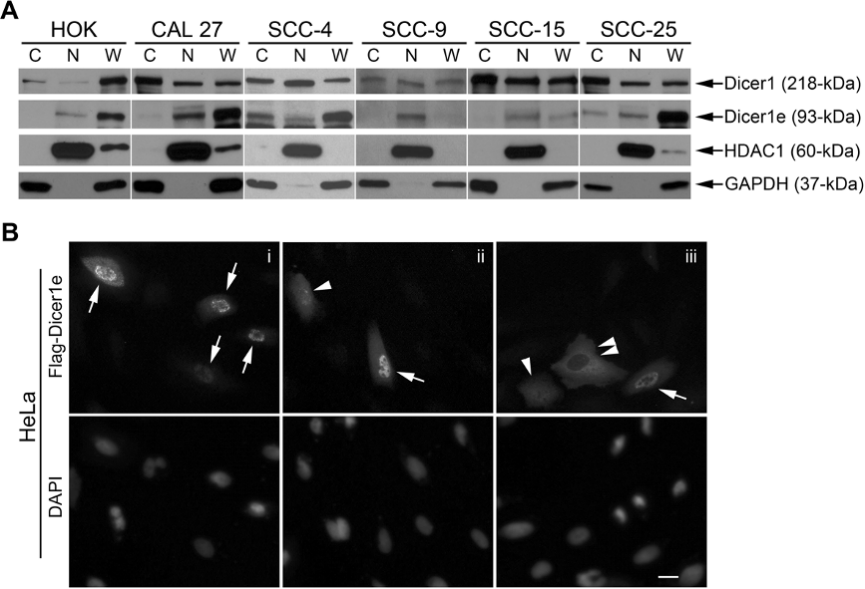
**The cellular localization of Dicer1e protein variant is predominantly nuclear. (A)** Western blot analysis of Dicer1 and Dicer1e protein levels in nuclear (N) and cytoplasmic (C) fractions and whole (W) cell lysates of human OSCC cell lines (CAL 27, SCC-4, SCC-9, SCC-15, and SCC-25) and normal HOKs. The histone deacetylase class 1 (HDAC1) and GAPDH were used as nuclear and cytoplasmic markers, respectively. **(B)** HeLa cells transiently transfected with FLAG-Dicer1e. Representative images are shown of recombinant Dicer1e localization in cells (upper panels i-iii). Cells with predominantly nuclear (arrows), nuclear and cytoplasmic (arrowheads), or cytoplasmic (double-arrowheads) Dicer1e localizations are indicated. Nuclei were counterstained with DAPI (lower panels i-iii). Scale bar: 20 μm.

Based on the NCBI AceView database, PSORT II analysis (http://psort.hgc.jp/) of the Dicer1e protein sequence predicts a possible bipartite NLS between amino acids 247 to 264 (KKVSNCNLYRLGKKKGL) and the subcellular location of Dicer1e protein to be most likely in the nucleus. Furthermore, a more recent study by Doyle *et al.* [41] found that the dsRBD of human Dicer1 functions as a NLS, a domain that is also present in Dicer1e. Therefore, to investigate the possibility that the Dicer1e protein was localized to the nucleus, we performed biochemical fractionation studies using cytoplasmic and nuclear protein extracts obtained from all the OSCC cell lines and HOKs (Figure 4A). Analyses of the data demonstrated that Dicer1e was localized to the nuclear fraction in all cell types examined. No differences in cellular localization were detected between the oral cancer cell lines and HOKs. However, in the OSCC cell lines exhibiting high expression levels of Dicer1e (CAL 27, SCC-4, and SCC-25), Dicer1e was also detected in the cytoplasm. Interestingly, the Doyle *et al.* study [41] found that a C-terminal fragment of Dicer1 (containing both RNase III domains plus the dsRBD), a construct structurally similar to Dicer1e (except that it lacked the N-terminal 210 amino acids present within the Dicer1e protein sequence), could localize to the cytoplasm, but failed to localize to the nucleus of HeLa cells. In an effort to resolve this discrepancy and to confirm our observed nuclear localization of Dicer1e, the localization of a recombinant FLAG-tagged Dicer1e protein was subsequently tested in transiently transfected HeLa cells (Figure 4B). Indirect immunofluorescent (IIF) analyses of these transfected cells confirmed nuclear localization of the recombinant Dicer1e protein (Figure 4B, upper panels i-iii, arrows), with nuclei verified by DAPI staining (Figure 4B, lower panels i-iii). Additionally, the recombinant Dicer1e protein was also found to be either equally distributed between both nuclear and cytoplasmic compartments (Figure 4B, upper panels ii and iii, arrowheads) or exclusively localized within the cytoplasm of transfected cells (Figure 4B, upper panel iii, double-arrowhead). Together, these data demonstrated that Dicer1e could primarily localize to the nucleus in cells, especially in low Dicer1e expressing cells (HOKs, SCC-9, and SCC-15) with the ability to also accumulate in the cytoplasm, particularly in high Dicer1e expressing cells (CAL 27, SCC-4, and SCC-25) or in transfected HeLa cells overexpressing a recombinant form of the Dicer1e protein. Moreover, these data suggested that the 210 amino acid sequence N-terminal of the first RNase III domain within Dicer1e appeared to be important for enabling the nuclear accumulation of Dicer1e.

### Depletion of Dicer1e expression inhibits the cell proliferation and clonogenic potential of oral cancer cell lines

**Figure 5:**
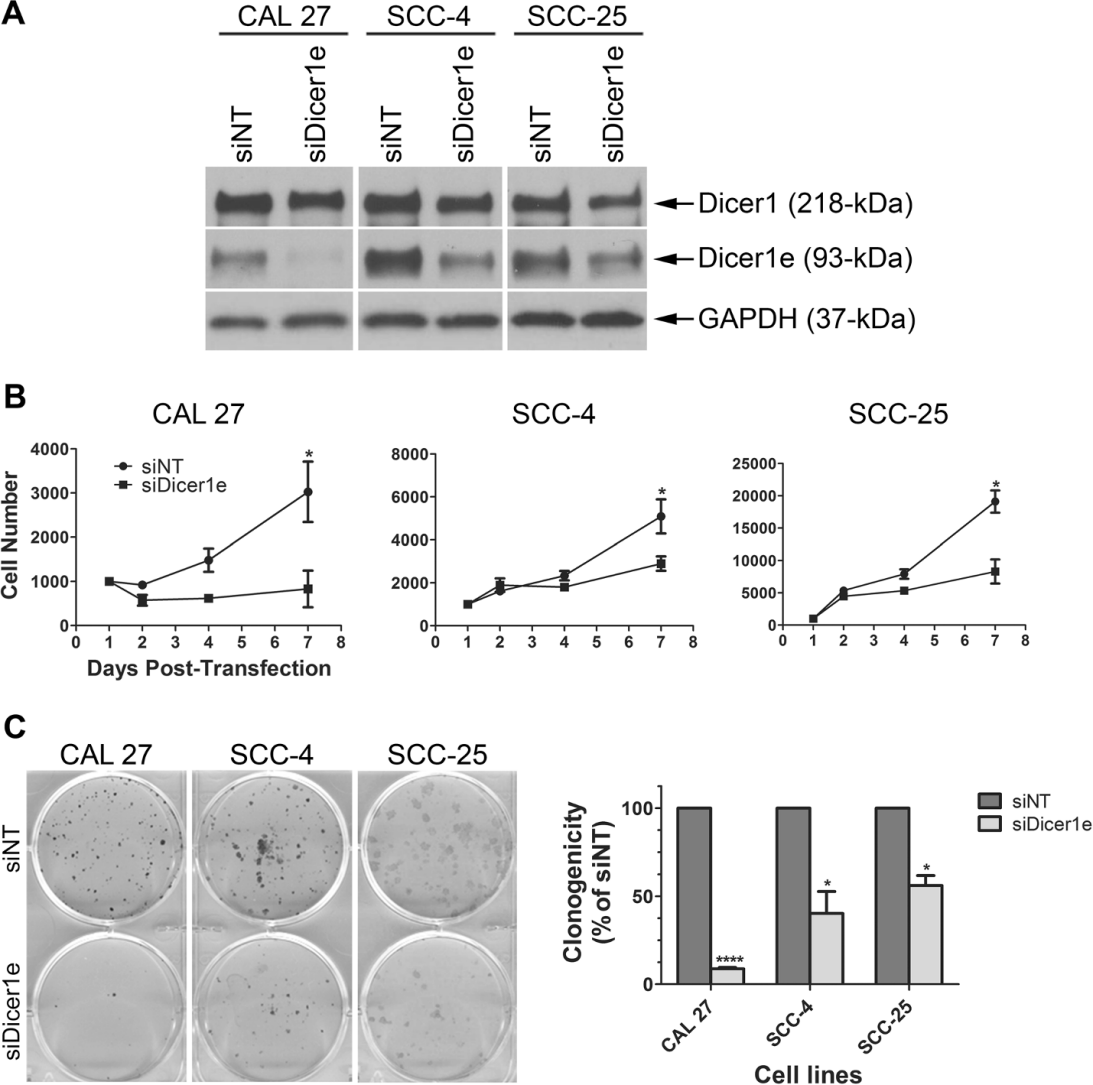
**Knockdown of Dicer1e impairs cell proliferation and clonogenicity of oral cancer cells. (A)** Western blot analysis of Dicer1 and Dicer1e protein levels in human OSCC cell lines (CAL 27, SCC-4, and SCC-25) 48 hours post-transfection with either control non-targeting siRNA (siNT) or siRNA targeting Dicer1e (siDicer1e). GAPDH was used as a loading control. **(B)** Cell proliferation assay was performed in OSCC cell lines CAL 27, SCC-4, and SCC-25, after treatment with siDicer1e compared to control siNT-treated cells. Data are mean ± SEM of three separate experiments performed in triplicate, where *P < 0.05 compared to siNT treated cells (Student’s *t* Test). **(C)** Assessment of clonogenic potentials of the siDicer1e-treated oral cancer cell lines CAL 27, SCC-4, and SCC-25, compared to control siNT-treated cells. The number of colonies were counted and the data are presented as mean ± SEM of three separate experiments performed in duplicate, where ****P < 0.0001, *P < 0.05 compared to siNT treated cells (Student’s *t* Test).

To determine whether the higher levels of Dicer1e contribute to the proliferation and clonogenic potential of oral cancer cells, we employed siRNA knockdown of Dicer1e expression. Using an siRNA designed to specifically target a unique sequence in Dicer1e mRNA (siDicer1e), we found the Dicer1e protein levels to be considerably reduced (by at least 80%) compared to cells transiently transfected with a control non-targeting siRNA (siNT) 48 hours post-treatment (Figure 5A). Of note, the Dicer1e-silencing effect was also assessed 7 and 9 days post-transfection and observed to persist up to 9 days, with maximum silencing occurring 7 days post-transfection for several of the cell lines (see Additional file 1: Figure S1). To ensure the targeting specificity of siDicer1e, we also analyzed the protein levels of Dicer1 and found Dicer1 expression to be unaffected upon treatment with siDicer1e compared to siNT (Figure 5A and Additional file 1: Figure S1). Having demonstrated that siDicer1e was capable of suppressing Dicer1e protein levels, but not Dicer1, we next examined the effects of Dicer1e depletion on cancer cell proliferation. The cell proliferation experiment was carried out where CAL 27, SCC-4, and SCC-25 cells were either transfected with siNT or siDicer1e, after which cell numbers were assayed 2, 4, and 7 days post-transfection (Figure 5B). The growth curves showed that silencing of Dicer1e significantly inhibited cell proliferation over a period of 7 days in all treated oral cancer cell lines compared to control siNT-transfected cells. Furthermore, consistent with the cell proliferation assays, in colony formation assays the depletion of Dicer1e in CAL 27, SCC-4, and SCC-25 cells lead to a significant reduction in foci number compared to control siNT-treated cells (Figure 5C). Together, these data demonstrated that the upregulation of Dicer1e was a contributing factor towards the transforming phenotypes of oral cancer cells.

### Silencing of Dicer1e induces either apoptosis and/or cell cycle arrest in oral cancer cell lines

**Figure 6:**
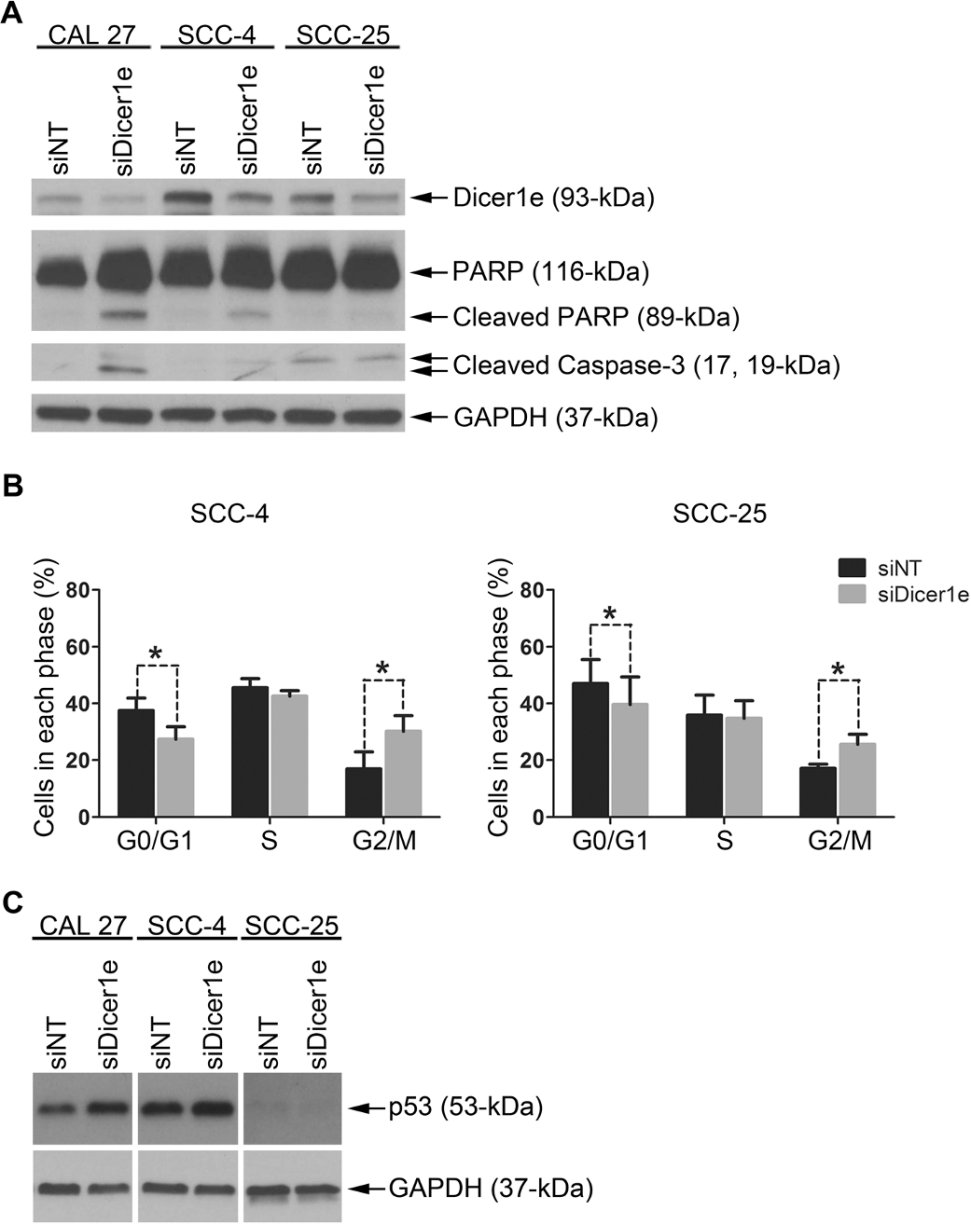
**Silencing of Dicer1e induces apoptosis and/or G2/M cell cycle arrest in oral cancer cells. (A)** Western blot analysis of PARP and caspase-3 cleavage levels in human OSCC cell lines (CAL 27, SCC-4, and SCC-25) 48 hours post-transfection with either control non-targeting siRNA (siNT) or siRNA targeting Dicer1e (siDicer1e). Dicer1e and GAPDH levels were monitored to ensure knockdown and equal loading of samples, respectively. **(B)** Cell cycle analyses of SCC-4 and SCC-25 cells after treatment with siDicer1e compared to control siNT-treated cells. Data are mean ± SEM of three independent experiments, where *P < 0.05 compared to siNT treated cells (Student’s *t* test). **(C)** Western blot analysis of p53 protein levels in CAL 27, SCC-4, and SCC-25 cell lines 48 hours post-transfection with either control siNT or siDicer1e. GAPDH was used as a loading control.

Because the depletion of Dicer1e had cytostatic effects on oral cancer cells, we next examined whether this inhibition in cell growth was possibly due to the induction of apoptosis and/or cell cycle arrest. To first assess the role of apoptosis, we transfected CAL 27, SCC-4, and SCC-25 cells with either siNT or siDicer1e, after which the cells were lysed 48 hours post-transfection and assayed for several apoptotic makers by Western blot analyses (Figure 6A). Examination of the different cellular lysates demonstrated that Dicer1e depletion resulted in a strong induction of apoptosis in CAL 27 cells, as was evident by the high levels of cleavage of caspase-3 and poly(ADP-ribose) polymerase (PARP), two caspase-dependent apoptotic markers. Very weak or no detectable changes in the cleavage of PARP and caspase-3 were observed in Dicer1e depleted SCC-4 and SCC-25 cells, respectively. Because of the weak or no apoptotic response in Dicer1e depleted SCC-4 and SCC-25 cells, we next examined whether Dicer1e silencing had any effect on the cell cycle progression of these two cell lines. Flow cytometry analyses demonstrated that depletion of Dicer1e in both cell lines affected their cell cycle distribution. As shown in Figure 6B, there was an increase in the percentage of cells in the G2/M phase 48 hours post-transfection with siDicer1e compared to control siNT-treated cells. More specifically, the percentage of cells at the G2/M phase significantly increased by approximately 13% and 10% in SCC-4 and SCC-25 cells, respectively. Thus, the above results indicated that suppression of Dicer1e levels in Dicer1e-overexpressing oral cancer cells could promote either apoptosis and/or cell cycle arrest.

The observed differences in cellular responses between these three oral cancer cell lines were most likely due to their molecular profile differences. For example, it is well documented that these three cell lines exhibit different types of mutations of the tumor suppressor protein p53: in particular, CAL 27 cells have a missense mutation in codon 193 of exon 6 (A → T transversion) [42, 43]; SCC-4 cells have a missense mutation in codon 151 of exon 5 (C → T transition) [44–46]; and SCC-25 cells have a frameshift mutation in codon 209 of exon 6 (two base pair deletion) that results in undetectable levels of p53 protein within these cells [44, 46, 47]. Because p53 is a key molecular regulator of apoptosis and cell cycle arrest [48], we examined the effects of Dicer1e silencing on p53 protein levels (Figure 6C). Upon silencing of Dicer1e in the different cell lines, it was found that CAL 27 cells that had the strongest induction levels of apoptosis had a large increase in p53 protein levels compared to control siNT treated cells, whereas the Dicer1e-depleted SCC-4 and SCC-25 cells that had either weak or no detectable apoptotic responses, had a moderate increase or no expression of p53, respectively. Thus, the apoptotic responses observed in the different oral cancer cell lines appeared to correlate with the degree of p53 protein levels that were induced upon Dicer1e depletion. Conversely, the cell cycle response appeared to function independently of p53, based on the fact that SCC-25 cells which lacked p53 were still able to undergo cell cycle arrest upon Dicer1e silencing.

### Down-regulation of Dicer1e sensitizes oral cancer cells to cisplatin

**Figure 7:**
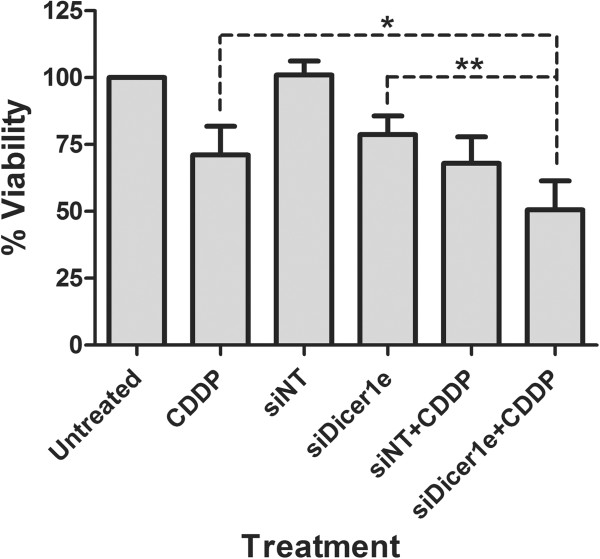
**Knockdown of Dicer1e enhances chemosensitivity to cisplatin in oral cancer cells.** The OSCC cell line SCC-25 was transfected with either control non-targeting siRNA (siNT) or siRNA targeting Dicer1e (siDicer1e). 24 hours post-transfection, 1.8 μM of cisplatin (CDDP) was added and 48 hours later the cell viability was assessed. Data are mean ± SEM of four independent experiments, where *P < 0.05 and **P < 0.01 compared to cisplatin and siDicer1e alone treated cells (Student’s *t* test).

Although several chemotherapeutic drugs, including cisplatin, have proven effective in the treatment of head and neck cancer [49–51], the failure of OSCCs to respond to chemotherapeutic treatments due to acquired resistance limits the overall success of these types of therapeutic strategies and in-part contributes to ~40% of oral cancer-related patient deaths [5, 52]. Therefore, to assess whether the depletion of Dicer1e could contribute towards the chemosensitization of oral cancer cells, we transfected SCC-25 cells with either siNT or siDicer1e 24 hours prior to the addition of 1.8 μM (IC_25_) of cisplatin for 48 hours. Of note, the IC_25_ value for cisplatin in SCC-25 cells was determined from a cell viability plot (Additional file 2: Figure S2). Analysis of cell viability between the different experimental treatments demonstrated that Dicer1e silencing in combination with cisplatin resulted in a significant reduction compared to cisplatin or siDicer1e alone (Figure 7). Combination treatments of siNT with cisplatin did not show any statistical differences in cell viability when compared with cisplatin alone. These data suggested that the enhanced response to cisplatin was associated with Dicer1e depletion in oral cancer cells.

## Discussion

The 93-kDa Dicer1e protein variant was first described in a study by Hinkal *et al.* [36], where it was reported to be differentially expressed in epithelial and mesenchymal breast cancer cells. In our study, Dicer1e was similarly found to be differentially expressed in oral cancer cells and during EMT, with epithelial oral cancer cells exhibiting higher levels of Dicer1e expression compared to cells of mesenchymal phenotype. Moreover, Dicer1e protein levels were found to be overexpressed in epithelial OSCC cell lines and in OSCC tissues compared to normal HOKs and adjacent normal tissues, respectively. Together, these data implied that the upregulation of Dicer1e expression potentially contributes towards the cellular transformation of normal oral epithelial cells with expression decreasing upon EMT and the development of more aggressive metastatic disease.

Although several studies had predicted that the 93-kDa Dicer1e protein variant was the product of a Dicer1 mRNA variant based on information available through the NCBI AceView database [23, 36], our study is the first to provide biochemical evidence for the existence of the Dicer1e transcript (2,822 nucleotides) via our 5′ and 3′-RACE analyses. The Dicer1e transcript we identified encoded an 820 amino acid truncated form of Dicer1 protein with a predicted M.W. of 93-kDa that comprised both RNase III domains and the dsRBD. Interestingly, the Dicer1e protein sequence was predicted to contain a NLS (247 – 264 aa) and our subcellular fractionation and IIF studies confirmed Dicer1e localization to the nucleus. A recent study by Doyle *et al.* [41], however, reported that this predicted NLS could not drive the nuclear localization of a reporter gene in HeLa cells. Instead, the study found that the dsRBD of human Dicer1 functioned as a NLS and that deletion of the N-terminal helicase domain was necessary to cause partial accumulation of Dicer1 in the nucleus upon leptomycin B treatment [41]. The significance of these findings is that Dicer1e lacks the helicase domain, but retains the dsRBD NLS which could account for its ability to localize to the nucleus. In addition to its nuclear localization, we also found Dicer1e to localize to the cytoplasm of several epithelial cancer cell lines (CAL 27, SCC-4, and SCC-25), where Dicer1e was found to be upregulated, and in transfected HeLa cells overexpressing a recombinant form of the Dicer1e protein. One possibility for this accumulation in the cytoplasm could have been due to the saturation of a nuclear transport system responsible for importing Dicer1e into the nucleus. Another possibility is that high expression levels of Dicer1e potentially perturbed its RNA-binding status, and hence its subcellular localization. Interestingly, the Doyle *et al.* study [41] found that when the RNA-binding potential of the RNase III domains were compromised within a C-terminal construct of Dicer1 (containing both RNase III domains plus dsRBD), a construct structurally similar to Dicer1e, it caused the protein to accumulate in the nucleus. Conversely, if one recalls, the wild-type construct failed to localize to the nucleus and instead accumulated in the cytoplasm, thus, suggesting that associated RNAs were masking the dsRBD NLS [41]. Consequently, the subcellular localization of Dicer1e may in-part depend on its RNA-binding status, which has the ability to mask/expose the dsRBD NLS and if dysregulated could lead to mislocalization of the protein.

Due to the fact that Dicer1e protein was increased in OSCC cell lines of epithelial phenotype and in OSCC tissues, the biological consequence of Dicer1e in oral cancer cells was examined, in particular its role in cell proliferation and clonogenic potential. The transfection of an exogenous siRNA targeting Dicer1e specifically, but not Dicer1, significantly inhibited the proliferation and clonogenic potential of three separate oral cancer cell lines, by either inducing apoptosis and/or G2/M cell cycle arrest. Moreover, the intensity of the apoptotic response appeared to correlate with the levels of p53 protein induced upon Dicer1e depletion. Interestingly, p53 levels have been found to determine the extent of an apoptotic response in cells [53, 54]. Regardless, these data demonstrated that Dicer1e contributes towards cancer cell growth and that its downregulation induces cellular stresses that result in either cell death and/or growth arrest. Furthermore, because Dicer1e silencing affected cell cycle kinetics, this suggested that its knockdown could enhance the sensitivity of a cancer cell to the effects of a DNA-damaging agent, such as cisplatin. In fact, in our studies, we found that Dicer1e silencing could indeed enhance the chemosensitivity of cancer cells to cisplatin, thus, implying that targeting of Dicer1e could be an effective strategy to sensitizing oral cancer cells to chemotherapeutics.

Finally, it is interesting to note, that a report studying the coordinated activities of human Dicer1 domains in RNA processing found that a truncated construct of Dicer1, termed hDcr-C, which is structurally similar to Dicer1e, was capable of binding and cleaving both long dsRNA and pre-miRNAs, with the binding/cleavage being more active towards dsRNAs [55]. This is particularly intriguing in light of the fact that a recently identified truncated form of Dicer1 in *C. elegans* termed, sDCR-1, that shares the same domains as hDCr-C and Dicer1e was found to enhance the exogenous RNAi pathway via its calatytic activity, whereas its concurrent role as a negative regulator of miRNA biogenesis functioned independently of this activity [56]. The enhanced exogenous RNAi response by sDCR-1 was postulated to be due to the loss of the helicase domain [56], which has been previously reported to have an autoinhibitory function that hinders the catalytic efficiency of human Dicer1 in processing long dsRNAs [57]. In line with this, a mouse oocyte-specific Dicer1 isoform lacking part of the helicase domain was similarly found to be more efficient at processing long dsRNA substrates compared to the full-length form [58]. As a result, due to the fact that Dicer1e shares the same domains as hDcr-C and sDCR-1, there is a strong likelihood that Dicer1e will similarly exhibit more activity towards the processing of perfectly matched dsRNAs. However, one cannot exclude the possibility that Dicer1e could also regulate miRNA biogenesis. In fact, in recognizing the structural similarities between sDCR-1 and Dicer1e, the Sawh and Duchaine study [56] also ectopically expressed Dicer1e in human cells and found that, similar to sDCR-1, it could modulate the biogenesis of specific miRNAs. Thus, based on these key pieces of evidence, the possibility that Dicer1e could have the ability to enzymatically process dsRNAs and modulate miRNA-specific biogenesis differently than Dicer1 is promising. Consequently, further studies will investigate the molecular function of Dicer1e in cells pertaining to RNAi/miRNA biology and its relevance to cancer.

## Conclusions

Our present study identified and characterized a 93-kDa Dicer1 protein variant, Dicer1e, in oral cancer cells. In particular, Dicer1e protein levels were found to be overexpressed in OSCC cell lines of epithelial phenotype and in OSCC tissues with its levels downregulated during EMT. Moreover, Dicer1e was encoded by a ~3 kbp transcript and was localized predominantly in the nucleus of both normal and cancer cells. Transient transfection of a recombinant form of Dicer1e also confirmed its localization within the nucleus. However, cytoplasmic localization was also detected in cancer cells exhibiting high levels of Dicer1e expression and in transfected cells that overexpressed the recombinant form of Dicer1e. Depletion of Dicer1e inhibited the cell proliferation and clonogenic potential of oral cancer cells by inducing either apoptosis and/or G2/M cell cycle arrest. In addition, Dicer1e silencing chemosensitized oral cancer cells to cisplatin treatment. Together, these data imply that Dicer1e upregulation contributes to oral cancer progression and that silencing its expression using RNAi strategies could be potentially used in conjunction with chemotherapeutic agents to curb the proliferation of cancer cells.

## Methods

### Cell culture

Human tongue SCC cell lines CAL 27, SCC-4, SCC-9, SCC-15, and SCC-25 were purchased from American Type Culture Collection (ATCC, Manassas, VA) and cultured in ATCC-specified complete growth media. Normal HOKs were obtained from ScienCell™ Research Laboratories (Carlsbad, CA) and cultured in oral keratinocyte medium supplemented with oral keratinocyte growth supplement and penicillin/streptomycin solution (ScienCell™ Research Laboratories). The HeLa cell line and the breast cancer cell line T47D were kindly provided by Gunhild Sommer (Medical University of South Carolina (MUSC)) and Yusuf Hannun (Stony Brook University), respectively, and were cultured in Dulbecco’s Modified Eagle Medium (Mediatech, Inc., Manassas, VA) supplemented with 10% fetal bovine serum (HyClone Laboratories Inc., Logan, Utah). All cells were maintained in a 37°C incubator with 5% CO_2_. To examine the effects of TGF-β treatment, SCC-4 and SCC-25 cells were grown to 40% confluency, after which they were serum-starved and then induced with TGF-β2 (a kind gift from Philip Howe (MUSC)), at 5 ng/ml for 8 days. Culture medium was changed every other day.

### Western blot analysis

Proteins were extracted as described in Cantini *et al*., [59]. Between 5-20 μg of the cell lysates were then resolved in Mini-PROTEAN^®^ TGX™ Precast 10% or 4-15% gels (BIO-RAD, Hercules, CA) and transferred to a PVDF membrane (Millipore, Bedford, MA). The PVDF membrane was blocked in 5% non-fat dried milk in Tris-HCl-buffered-saline supplemented with 0.1% Tween-20 (TBS-T) for 2 hours at room temperature (RT). Afterwards, the membrane was incubated overnight at 4°C with either of the following primary antibodies: anti-Dicer1 (1:1,000, Abcam, Cambridge, MA), an antibody also capable of detecting Dicer1d/e [23, 36], anti-E-cadherin (1:2,500, BD Biosciences, San Jose, CA), anti-vimentin (1:1,000, Santa Cruz Biotechnology, Santa Cruz, CA), anti-cleaved Caspase-3 (1:1,000, Cell Signaling Technology, Danvers, MA), anti-GAPDH (1:10,000, Cell Signaling Technology), anti-HDAC1 (1:10,000, Cell Signaling Technology), anti-β-actin (1:12,000, Sigma, Saint Louis, MO), anti-p53 (1:1,000, Dako, Carpinteria, CA), and anti-PARP (1:1,000, Cell Signaling Technology). Subsequently, the membranes were washed four times with TBS-T and then incubated with the corresponding secondary antibodies either horseradish peroxidase-conjugated goat anti-mouse (1:2,000 or 1:20,000) or anti-rabbit IgG (1:2,000 or 1:20,000, SouthernBiotech, Birmingham, AL) for 1 hour at RT. Immunoreactive bands were detected with the SuperSignal Chemiluminescent system (Thermo Fisher Scientific, Waltham, MA), according to the manufacturer’s instructions. Quantitative determinations were performed by densitometry of the corresponding non-saturated band intensities, normalized against the respective intensities of GAPDH using ImageJ software [60].

### Human tissue extracts

Frozen human tongue SCC tissue with adjacent normal tissue samples were obtained from the MUSC Hollings Cancer Center (HCC) Biorepository & Tissue Analysis Shared Resource. The study was approved by the ethics committee of the MUSC Institutional Review Board. Patients’ identities associated with all the tissue samples were removed prior to analyses. After procurement of the tissues, the samples were homogenized with stainless steel blend beads (Next Advance, Averill Park, NY) in lysis buffer containing 100 mM Tris-HCl, pH 7.4, 2% SDS, and 1× protease inhibitor cocktail (Thermo Fisher Scientific) using a Next Advance Bullet Blender^®^, according to the manufacturer’s instructions. Afterwards, 10 μg of protein extracts were used for Western blot analysis.

### RNA extraction and 5′-RACE

SCC-25 cells were used to isolate mRNA with the FastTrack^®^ 2.0 kit (Life Technologies, Grand Island, NY), following the manufacturer’s protocol. Once purified, 250 ng of the mRNA were then used to perform 5′-RACE with the GeneRacer^®^ kit (Life Technologies) following the manufacturer’s instructions. Briefly, the mRNA was dephosphorylated and decapped before an RNA oligo of known sequence was ligated to the 5′-end. Subsequently, a reverse transcription reaction was carried out using an oligo dT primer. Afterwards, PCR amplification was performed using the kit’s GeneRacer 5′-sense primer (GR5S) and a custom made Dicer1e-specific antisense primer (Dic1e5AS, 5′-TTCAGCTAAAATCCGCAGGAAGTGAT-3′). The PCR parameters used were as follows: 94°C for 2 minutes (1 cycle); 94°C for 30 seconds and 72°C for 3 minutes (5 cycles); 94°C for 30 seconds and 70°C for 3 minutes (5 cycles); 94°C for 30 seconds, 66°C for 30 seconds, and 68°C for 3 minutes (25 cycles); 68°C for 10 minutes (1 cycle). After PCR, the amplification product was electrophoresed on a 2% agarose gel and the predicted PCR band was gel purified using the QIAquick^®^ PCR Purification kit (Qiagen, Valencia, CA). A second PCR was performed using the same GR5S and Dic1e5AS primers to increase the PCR fragment copy number in order to facilitate cloning using the TOPO^®^ TA Cloning^®^ kit (Life Technologies). Colonies were screened for insert by restriction enzyme digestion, after which purified plasmid DNAs from positive colonies were sent for sequencing.

### 3′-RACE

50 ng of purified mRNA from SCC-25 cells was used to perform 3′-RACE with the FirstChoice^®^ RLM-RACE kit (Life Technologies), following the manufacturer’s protocol. Briefly, a reverse transcription reaction was carried out with the kit’s 3′-RACE Adapter containing a string of T residues to prime the reaction. Afterwards, PCR was carried out with the kit specific 3′-RACE outer antisense primer and a Dicer1/1e-specific sense primer, (Dic1/1e3S1, 5′-GGTTCCAGAACTCTGTGCTA-3′). The PCR parameters used were as follows: 94°C for 5 minutes (1 cycle); 94°C for 45 seconds, 60°C for 30 seconds, 72°C for 45 seconds (35 cycles); 72°C for 7 minutes (1 cycle). Subsequently, a nested PCR was performed with 1 μl of initial PCR product, using the kit’s 3′-RACE inner antisense primer and a nested Dicer1e-specific sense primer (Dic1e3S2, 5′-CACTTCCTGCGGATTTTAGCTGAA-3′). The same PCR parameters were used as for the initial PCR. After the nested PCR, the amplification product was gel purified and cloned, as described above, after which purified plasmid DNAs from positive colonies were sent for sequencing.

### DNA sequencing

Sequencing was performed by Eurofins MWG Operon (Huntsville, AL). M13 forward and reverse primers were used to sequence the complete sense and antisense strands of the 5′-RACE product and part of the 3′-RACE product. An additional series of sequencing primers were designed to sequence the complete sense and antisense strands of the 3′-RACE product. For the sense strand the primers used were: F-SeqII, 5′-CCTTTTTAAAGCATGCCATC-3′; F-SeqIII, 5′-AGCAGTCCATTTCTTACGAC-3′; and F-SeqIV, 5′-TCTCTCCTGAGCTCTTCCAT-3′. For the antisense strand the primers used were: R-SeqII, 5′-ATGGAAGAGCTCAGGAGAGA-3′; R-SeqIII, 5′-GTCGTAAGAAATGGACTGCT-3′; and R-SeqIV, 5′-GATGGCATGCTTTAAAAAGG-3′. Upon receiving the sequencing data, Vector NTI^®^ (Life Technologies) software was used to assemble contigs in order to obtain the complete Dicer1e sequence.

### Custom gene synthesis and cloning

After verifying the Dicer1e sequence, the gene was custom synthesized by GenScript (Piscataway, NJ) from the start to stop codons with *NotI* and *ApaI* restriction sites added 5′ and 3′ relative to each codon, respectively. The full-length gene was then cloned by GenScript into the *EcoRV* site within the pUC57 plasmid vector, after which it was subsequently subcloned in-frame into pGen2.1, a mammalian N-terminal FLAG-tag gene expression vector, using the *NotI* and *ApaI* restriction sites. All constructs were verified by DNA sequencing. Upon receiving the the pGen2.1-Dicer1e plasmid construct, the contruct was transformed into NEB 5-α competent *E. coli* cells (New England BioLabs, Ipswich, MA) and purified for transfection purposes using the EndoFree^®^ Plasmid Maxi kit (Qiagen).

### Biochemical fractionation

HOK and OSCC cell lines were plated onto 6-cm dishes and grown to 80% confluency. Afterwards, the cells were trypsinized, centrifuged at 500 × *g* for 5 minutes, washed with ice-cold 1× PBS and then centrifuged again. Cytoplasmic and nuclear extracts were obtained from the cell pellets using the NE-PER^®^ kit (Thermo Fisher Scientific), according to the manufacturer’s instructions. 4.5 μg of cytoplasmic and nuclear protein extracts were used for Western blot analysis.

### Plasmid transfections and immunofluorescence

The pGen2.1-Dicer1e plasmid construct was transiently tranfected into HeLa cells grown on Collagen Type I coated 8-chamber slides (BD Biosciences) using Lipofectamine^®^ 2000 (Life Technologies), according to the manufacturer’s recommendation. 24 hours post-transfection the cells were processed for indirect immunofluorescence as previously described [59] using anti-FLAG^®^ M2 antibody (1:100, Sigma). Alexa Fluor^®^ 488-conjugated goat anti-mouse IgG (1:400, Life Technologies) was used as the corresponding secondary antibody. Images were obtained using a Zeiss (Thornwood, NY) Axio Observer.D1 microscope equipped with a LD A-Plan × 20/0.3 Ph1 objective.

### siRNAs and transfections

The control siGENOME Non-Targeting siRNA #5 (siNT) and the siRNA targeting the unique sequence of Dicer1e (siDicer1e, sense: 5′-CCUGCGGAUUUUAGCUGAAdTdT-3′, antisense: 5′-UUCAGCUAAAAUCCGCAGGdTdT -3′) were synthesized by Thermo Fisher Scientific Dharmacon (Lafayette, CO). Unless specified differently, 50 nM of either control siNT or siDicer1e were transiently transfected into CAL 27, SCC-4, and SCC-25 cells using INTERFERin^®^ (Polyplus-transfection, New York, NY), according to the manufacturer’s recommendations.

### Cell proliferation assay

Cell proliferation was determined using the CyQUANT^®^ Cell Proliferation Assay Kit (Life Technologies). Briefly, 100,000 cells were seeded on 12-well plates one day prior to treatment. Afterwards, the cells were transiently transfected with either control siNT or siDicer1e. 24 hours post-transfection, 1000 cells were re-seeded onto black 96-well plates with clear bottom and then 2, 4, and 7 days post-transfection the number of cells were assayed, according to the manufacturer’s instructions. Fluorescence at 480/520 nm was measured using a BioTek (Winooski, VT) Synergy HT plate reader.

### Colony formation assay

24 hours post-treatment with the specified siRNAs, the OSCC cell lines were reseeded at 500 or 1,000 cells per well on 6-well plates. After eleven days of incubation, the cells were fixed in an acetic acid:methanol solution (1:7 ratio), and then stained with Giemsa. Colonies with more than 50 cells were counted.

### Flow cytometry

Cell cycle analysis was determined by analyzing the DNA content of the cells using propidium iodide (PI), as described by Calipel *et al*. [61] with only minor modifications. Briefly, 48 hours post-treatment, the cells were trypsinized, washed in 1× PBS, and then fixed in ice-cold 70% ethanol at 4°C overnight. Afterwards, the cells were rehydrated in ice-cold 1× PBS, centrifuged at 500 × *g* for 5 minutes, resuspended in 0.5 ml of PI/RNase Staining Solution (Cell Signaling Technology), and stained at 4°C for at least 2 hours. Flow cytometric analyses of the samples were then performed using a BD FACSCalibur Analytical Flow Cytometer available through the MUSC HCC Flow Cytometry & Cell Sorting Shared Resource. Data analyses were performed using ModFit LT software.

### Drug sensitivity assay

The drug sensitivity was determined using the CellTiter 96^®^ AQueous One Solution Cell Proliferation Assay (Promega, Madison, WI). Briefly, 3,000 cells were seeded on 96-well plates one day prior to treatment. Afterwards, the cells were transiently transfected with 25 nM of either control siNT or siDicer1e. 24 hours post-transfection, the treatment was removed and fresh culture medium containing 1.8 μM of cisplatin was added (Sigma). 48 h post-treatment with cisplatin the cell viability was assayed, according to the manufacturer’s instructions. Absorbance at 490 nm was measured using a BioTek Synergy HT plate reader.

### Statistical analysis

The results from three or four independent experiments performed in either duplicate or triplicate were presented as mean ± SEM. Student’s *t* test was used for statistical evaluation. Statistical analyses were performed using GraphPad Prism 6 software (La Jolla, CA).

## Electronic supplementary material

Additional file 1: Figure S1: Assessment of long-term Dicer1e silencing in oral cancer cells. Western blot analysis of Dicer1 and Dicer1e protein levels in human OSCC cell lines (CAL 27, SCC-4, and SCC-25) 7 and 9 days post-transfection with either control non-targeting siRNA (siNT) or siRNA targeting Dicer1e (siDicer1e). β-actin was used as a loading control. (JPEG 660 KB)

Additional file 2: Figure S2: Dose response of SCC-25 oral cancer cells to cisplatin. Cells were treated with increasing concentrations of cisplatin (ranging from 0.001 to 30 μM), after which cell viability was assayed 48 hours post-treatment. Data are mean ± SEM of three independent experiments. (JPEG 254 KB)

## Abbreviations

OSCCs: Oral squamous cell carcinomas
RNAi: RNA interference
dsRNAs: Double-stranded RNAs
miRNAs: microRNAs
siRNAs: Small interfering RNAs
Pre-miRNAs: Precursor miRNAs
RISC: RNA-induced silencing complex
SCC: Squamous cell carcinoma
HOK: Human oral keratinocytes
TBS-T: Tris-HCl-buffered-saline supplemented with 0.1% Tween-20
RT: Room temperature
MUSC: Medical University of South Carolina
HCC: Hollings Cancer Center
GR5S: GeneRacer 5′-sense primer
Dic1e5AS: Dicer1e-specific antisense primer
Dic1/1e3S1: Dicer1/1e-specific sense primer
Dic1e3S2: Dicer1e-specific sense primer
siNT: siGENOME Non-Targeting siRNA #5
siDicer1e: siRNA targeting the unique sequence of Dicer1e
PI: Propidium iodide
NLS: Nuclear localization signal
dsRBD: dsRNA binding domain
PARP: Poly(ADP-ribose) polymerase
EMT: Epithelial-mesenchymal transition
DAPI: 4′,6-diamidino-2-phenylindole
IIF: Indirect immunofluorescent.
