# Supplementary figures and images for: Identification and characterization of Dicer1e, a Dicer1 protein variant, in oral cancer cells
Source: Mol Cancer. 2014 Aug 13;13:190. doi: 10.1186/1476-4598-13-190 (PMC4141963; doi:10.1186/1476-4598-13-190)

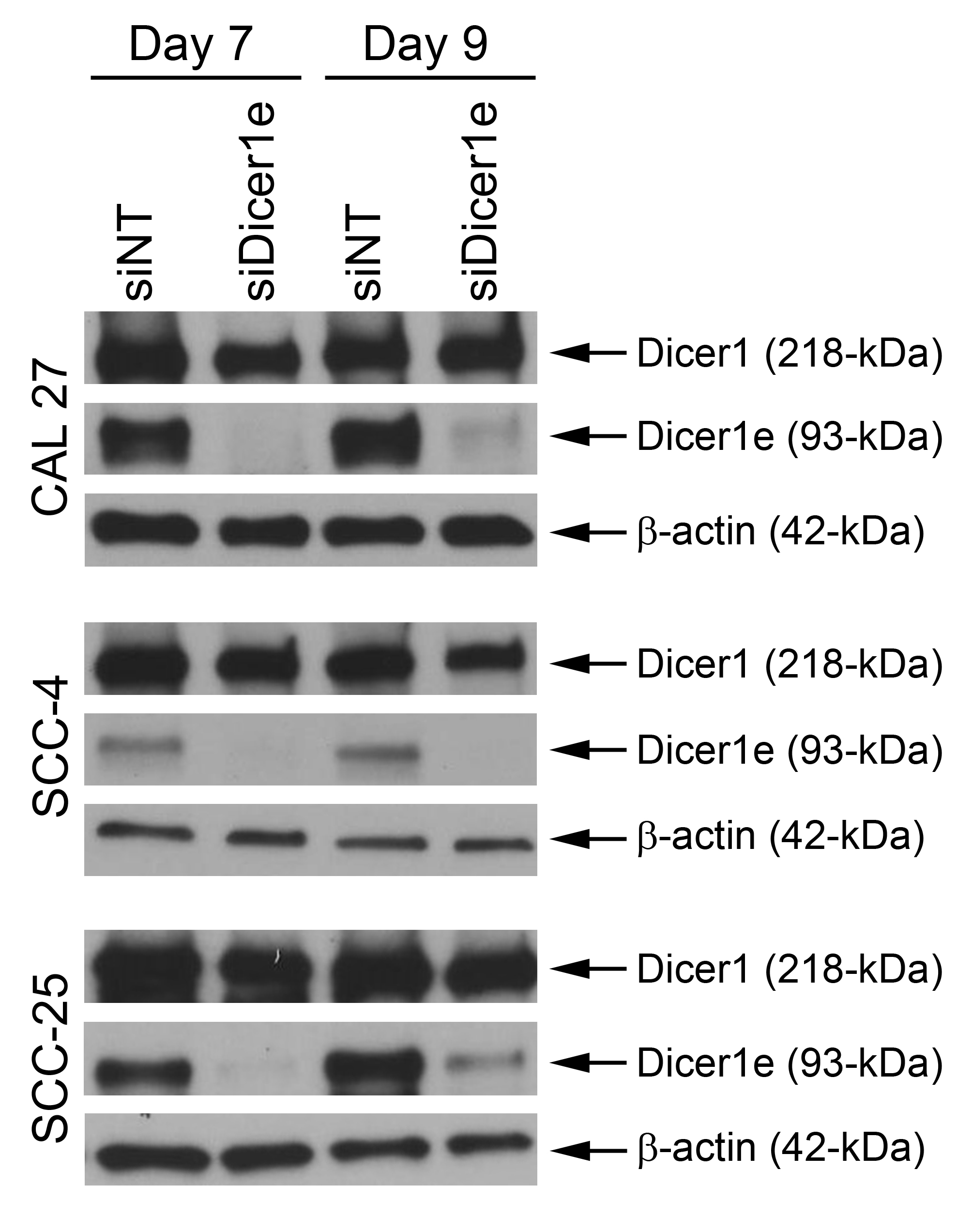

Supplement: Supplementary file 1 — Additional file 1: Figure S1: Assessment of long-term Dicer1e silencing in oral cancer cells. Western blot analysis of Dicer1 and Dicer1e protein levels in human OSCC cell lines (CAL 27, SCC-4, and SCC-25) 7 and 9 days post-transfection with either control non-targeting siRNA (siNT) or siRNA targeting Dicer1e (siDicer1e). β-actin was used as a loading control. (JPEG 660 KB) [file 12943_2014_1393_MOESM1_ESM.jpeg]

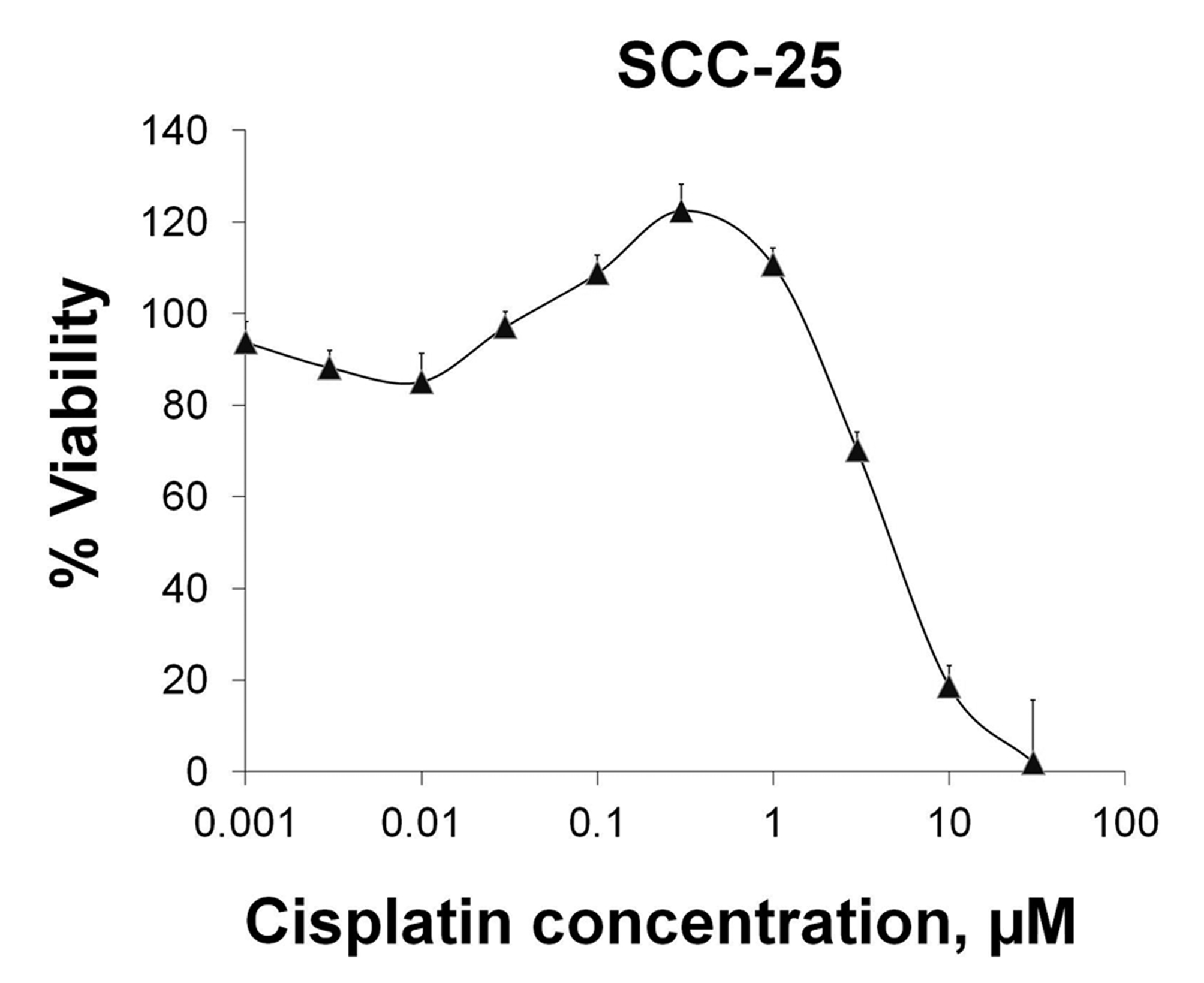

Supplement: Supplementary file 2 — Additional file 2: Figure S2: Dose response of SCC-25 oral cancer cells to cisplatin. Cells were treated with increasing concentrations of cisplatin (ranging from 0.001 to 30 μM), after which cell viability was assayed 48 hours post-treatment. Data are mean ± SEM of three independent experiments. (JPEG 254 KB) [file 12943_2014_1393_MOESM2_ESM.jpeg]
